# Identifying the role of aging-related genes in intracranial aneurysms through bioinformatics analysis

**DOI:** 10.1186/s41016-025-00407-5

**Published:** 2025-10-07

**Authors:** Junlin Kang, Shilai Tian, Xiaofeng Xu, Gang Yang

**Affiliations:** 1 Department of Neurosurgery, The First Hospital of Lanzhou University, Lanzhou, China; 2 The First School of Clinical Medicine, Lanzhou University, Lanzhou, China

**Keywords:** Intracranial aneurysm, Aging, Bioinformatics analysis, Aging-related genes

## Abstract

**Background:**

Intracranial aneurysm(IA) are among the most common cerebrovascular diseases, and their rupture can lead to severe consequences. Aging plays a significant role in the onset and progression of many diseases, yet it remains understudied in the context of intracranial aneurysms. The aim of this study is to investigate the role of aging-related genes in the development of intracranial aneurysms using public databases, in order to understand the underlying biological mechanisms.

**Methods:**

Gene expression profiles for intracranial aneurysms were downloaded from the GEO database. Human aging-related genes were obtained from the HAGR website. Differentially expressed gene analysis and WGCNA were used to identify core hub genes. GO and KEGG enrichment analyses were conducted to determine the potential biological functions and pathways that these differentially expressed aging-related genes in intracranial aneurysms might be involved in. Based on the hub genes, co-expression gene networks and Gene-TF-miRNA regulatory networks were constructed. Further exploration of drug-gene interactions was conducted to screen potential target drugs.

**Results:**

Through the intersection of aging-related genes and differentially expressed genes in IA, 32 common differentially expressed genes were identified, with 20 genes upregulated and 12 genes downregulated. GO enrichment analysis showed that these genes were mainly involved in epithelial cell proliferation and regulation, peptide enzyme activity modulation, and metabolic Homeostasis. KEGG enrichment analysis showed that these genes were primarily involved in the adipocytokine signaling pathway, growth Hormone synthesis,secretion and action, neurotrophin signaling pathway, and longevity regulating pathway. WGCNA was used to identify genes highly correlated with the IA phenotype, and an intersection with the 32 differentially expressed aging-related genes yielded 11 candidate Hub DEARGs. The expression of the candidate Hub DEARGs was validated using an external dataset, ultimately confirming 4 hub DEARGs related to intracranial aneurysms. Among them, NGFR and ADCY5 were downregulated, while BUB1B and SERPINE1 were upregulated.

**Conclusions:**

This study identified four aging-related genes, NGFR, ADCY5, SERPINE1, and BUB1B, that are associated with intracranial aneurysms. This provides new insights into the molecular mechanisms underlying the development of intracranial aneurysms. The identified core genes provide promising leads for further experimental research to explore the pathogenesis of the disease.

## Background

Intracranial aneurysm (IA) is among the most common and dangerous cerebrovascular diseases. It is formed due to the combined effects of hemodynamics, genetic factors, and environmental factors. IA is a pathological abnormal bulge in the intracranial vascular wall, characterized by changes in local vascular wall structure and tissue remodeling [1]. Rupture of an IA can cause aneurysmal subarachnoid hemorrhage, with a mortality rate as high as 66.7%, and survivors may suffer from severe neurological dysfunctions [2]. A meta-analysis involving 1450 cases of unruptured intracranial aneurysms in 94,912 patients from 21 countries found that older age is a significant risk factor for the development of IAs [3]. The risk of aneurysm rupture also increases with age. Therefore, aging-related genes may have a potential role in the occurrence of intracranial aneurysms.

Aging is an inevitable biological process characterized by cell-type and tissue-specific changes that can lead to many chronic and age-related pathological alterations [4]. Despite losing the ability to divide, aging cells remain active and metabolically competent. Aging plays a significant role at the genetic, molecular, and cellular levels [5]. In humans, cellular aging primarily affects endothelial cells, immune cells, and fibroblasts [6]. Excessive reactive oxygen species (ROS) are generated during aging, thereby reducing nitric oxide bioavailability and impairing endothelium-dependent vasodilation [7]. ROS also directly impair endothelial cells, thereby accelerating cellular senescence [8]. Senescent cells secrete senescence-associated secretory phenotype (SASP) factors, which generate pro-inflammatory cytokines (e.g., IL-6, TNF-α) [9], establishing a chronic inflammatory milieu that promotes monocyte adhesion and inflammatory infiltration of the vascular wall [10]. Oxidative stress and inflammation act synergistically to promote the phenotypic switching of vascular smooth muscle cells (VSMCs) from a contractile to a synthetic state, thereby inducing medial layer thickening and excessive collagen deposition in blood vessels [11]. Alterations in matrix metalloproteinase (MMP) activity induce degradation of elastic fibers, thereby exacerbating arteriosclerosis [12]. The functional decline of the NRF2 antioxidant pathway during aging compromises cellular defense capacity against oxidative damage [13]. Concurrently, upregulated expression of the NADPH oxidase (NOX) family generates a self-amplifying cycle of ROS production [14]. These pathological processes collectively drive the development of endothelial dysfunction, arteriosclerosis, and vascular remodeling within the vessel wall [15]. Aging-related genes play a crucial role in the initiation and regulation of cellular aging and affect vascular tissue in complex ways.

In order to gain a deeper understanding of the pathological characteristics and pathogenesis of intracranial aneurysms, and to explore the role of aging-related genes in them. Through bioinformatics analysis, we aim to reveal the potential role of aging-related genes in the occurrence of IA and their signaling pathways and molecular regulatory mechanisms. This provides valuable options and research directions for understanding the complex pathogenesis and early diagnosis of intracranial aneurysms. Additionally, this provides a basis for the development of therapeutic drugs for IA and potential noninvasive treatment modalities.

## Methods

### Acquisition and processing of intracranial aneurysm datasets

We searched for “intracranial aneurysm” in the Gene Expression Omnibus (GEO) database (http://www.ncbi.nlm.nih.gov/geo/), selected human tissue, and reviewed aneurysmal tissue samples from patients with intracranial aneurysms and normal vascular tissue samples. Three suitable datasets were selected, namely microarray datasets GSE75436, GSE54083, and GSE15629. The training set GSE75436 dataset includes 15 samples of unruptured intracranial aneurysm tissue and 15 samples of normal superficial temporal artery(STA) tissue. The validation set GSE54083 dataset includes 8 samples of ruptured intracranial aneurysm tissue, 5 samples of unruptured intracranial aneurysm tissue, and 10 samples of normal superficial temporal artery tissue. The validation dataset GSE15629 comprised tissue specimens from eight ruptured intracranial aneurysms, six unruptured intracranial aneurysms, and five middle meningeal arteries (MMA). Annotate gene expression profiles using GPL platform annotation files and convert gene probes into gene symbols. We clean the raw data by removing probes without gene annotations. If multiple probes correspond to the same gene symbol, we choose to retain the maximum expression value and perform log2 conversion and normalization on the data. From Human Aging Genomic Resources (HAGR; https://genomics.senescence.info/), download 307 genes related to human aging from the database [16].

### Identify differentially expressed genes (DEGs) and differentially expressed age-related genes (DEARGs) in IA

To classify the samples in the training set GSE75436 dataset into IA and STA groups, we use the “factoextra” R package (version 1.0.7) to perform principal component analysis and visualize the differences between the IA and STA groups. To perform differential gene expression analysis using the “limma” R package (version 3.60.2) [17]. To filter the DEGs based on the criteria of log2 |Fold Change|> 1 and adjusted p-value < 0.05. To obtain the differentially expressed aging-related genes (DEARGs) by intersecting the acquired DEGs with 307 aging-related genes (ARGs). To plot the distribution volcano plot of DEGs and heatmap of DEARGs using R software packages"ggplot2"(version 3.5.1) and"pheatmap"(version 1.0.12). To create a Venn diagram of the intersection between DEGs and ARGs using the EVenn online tool (http://www.ehbio.com/test/venn) [18].

### Enrichment analysis

Gene Ontology (GO) functional enrichment analysis primarily encompasses three aspects: biological processes, cellular components, and molecular functions. The Kyoto Encyclopedia of Genes and Genomes (KEGG) pathway enrichment analysis integrates gene functions and genomics as a whole network, studying gene functional annotation and gene pathway enrichment. To perform GO and KEGG enrichment analysis on DEARGs using the"ClusterProfiler"R package (version 4.12.0) [19]. To filter the results of GO and KEGG enrichment analysis on DEARGs using an adjusted p-value < 0.05 as the selection criterion. To visualize the results of enrichment analysis on DEARGs using the"ggplot2"R package (version 3.5.1).

### Weighted gene co-expression network analysis (WGCNA) and the identification of candidate hub genes

WGCNA is an important tool in bioinformatics analysis and has been widely applied in the study of phenotypic traits and gene association analysis. To conduct WGCNA analysis using the"WGCNA"R package (version 1.72–5) [20]. We constructed a coexpression network using all gene expression data from the GSE75436 dataset as input, with IA and STA as phenotypic trait data. Firstly, we used the hclust function to cluster the samples and remove outliers. Secondly, we determined the appropriate soft threshold to obtain a scale-free network. Thirdly, we used the “cutree Dynamic” feature to perform hierarchical clustering of genes and identify modules. The correlation between modules and IA and STA was represented using a heatmap. In the module with the highest correlation to IA, we plotted a scatterplot of gene significance (GS) and module membership (MM). To further filter out candidate hub DEARGs, we intersected the co-expressed genes in that module with DEARGs.

### External validation

To verify the reliability of the results, the expression levels of candidate hub DEARGs were further validated using an external dataset. We divided the samples in the validation set GSE54083 dataset into IA and STA groups to validate the differential expression of candidate core genes. Extract the expression levels of hub DEARGs from the GSE54083 dataset and use the Wilcoxon test to calculate and visualize the expression differences between the IA and STA groups. Samples from the GSE15629 validation dataset were stratified into an IA and MMA group, with comparative validation performed using identical methodology. The validated genes will be used as hub DEARGs for subsequent analysis.

### Interaction network and functional analysis of hub DEARGs co-expressed genes

To further explore the potential molecular mechanisms of hub DEARGs, we utilized the GeneMANIA database (http://www.genemania.org/) to construct a protein–protein interaction network for hub DEARGs and obtain co-expressed genes along with their biological functions [21]. To further perform GO enrichment analysis on these co-expressed genes.

### Analysis of immune cell infiltration in IA organization

Immune cell infiltration analysis was performed using CIBERSORT. Use the R package “CIBERSORT” (version 1.03) to analyze the immune cell components of intracranial aneurysm tissue and normal vascular controls.

### Constructing a gene-TF-miRNA network and drug-gene interactions

Use the NetworkAnalyst [22] comprehensive platform for visualizing gene expression analysis to predict transcription factors (TF) and miRNAs upstream of genes. Then, based on the hub DEARGs, we constructed a gene-TF-miRNA network.

Use DGIdb database (https://www.dgidb.org/) to predict drugs for hub DEARGs and screen potential drugs targeting aging-related genes [23]. To visualize the results, use the online platform RAWGraphs 2.0 (https://app.rawgraphs.io/).

### Statistical analysis

All statistical analyses were conducted using R software (version 4.4.0). Wilcoxon test was used to compare the differences in continuous variables between two groups. *P* < 0.05 indicates a statistically significant difference.

## Results

### Identification of DEGs and DEARGs in IA

**Fig. 1:**
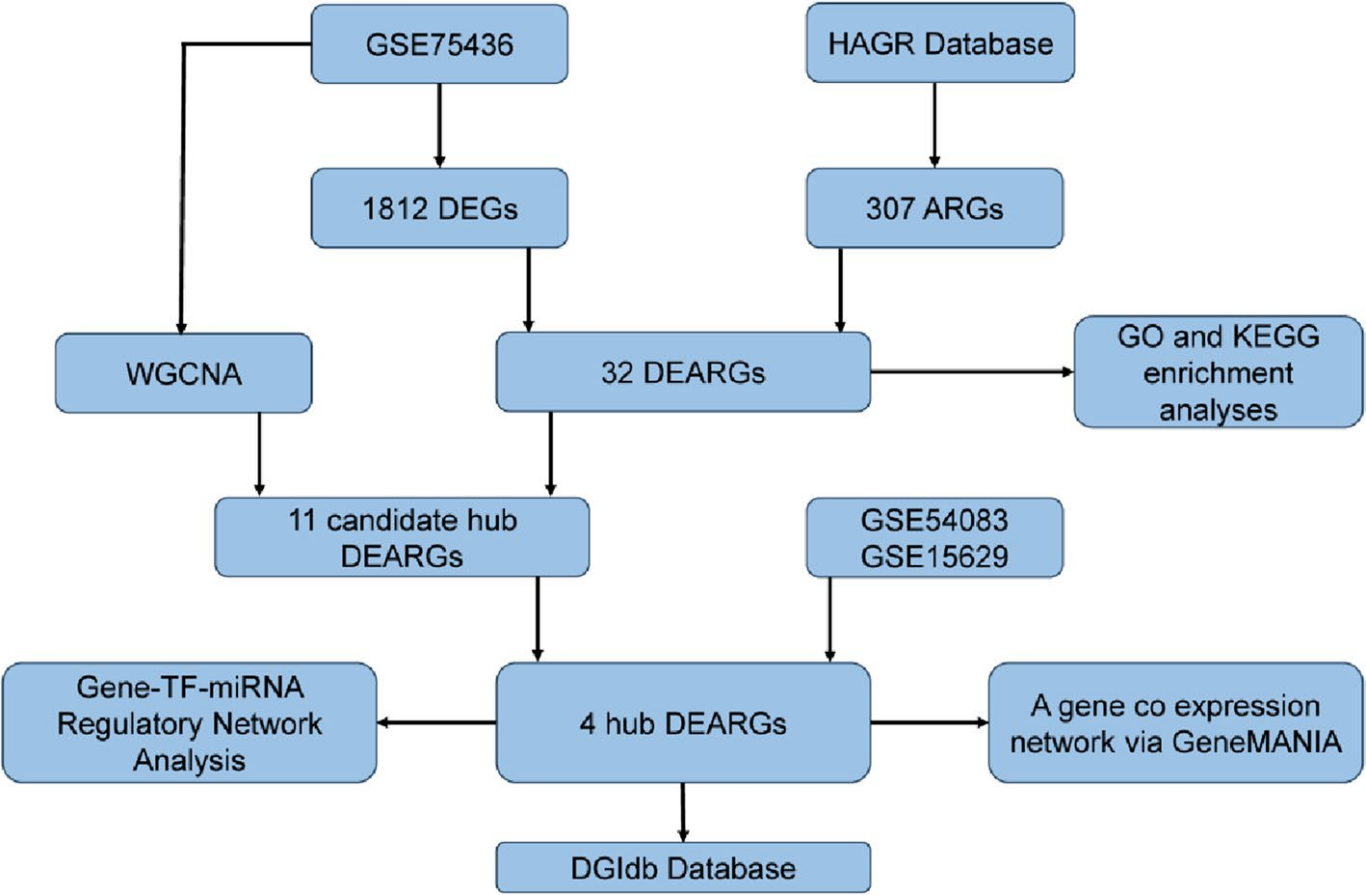
The flowchart for data analysis in this study

**Fig. 2:**
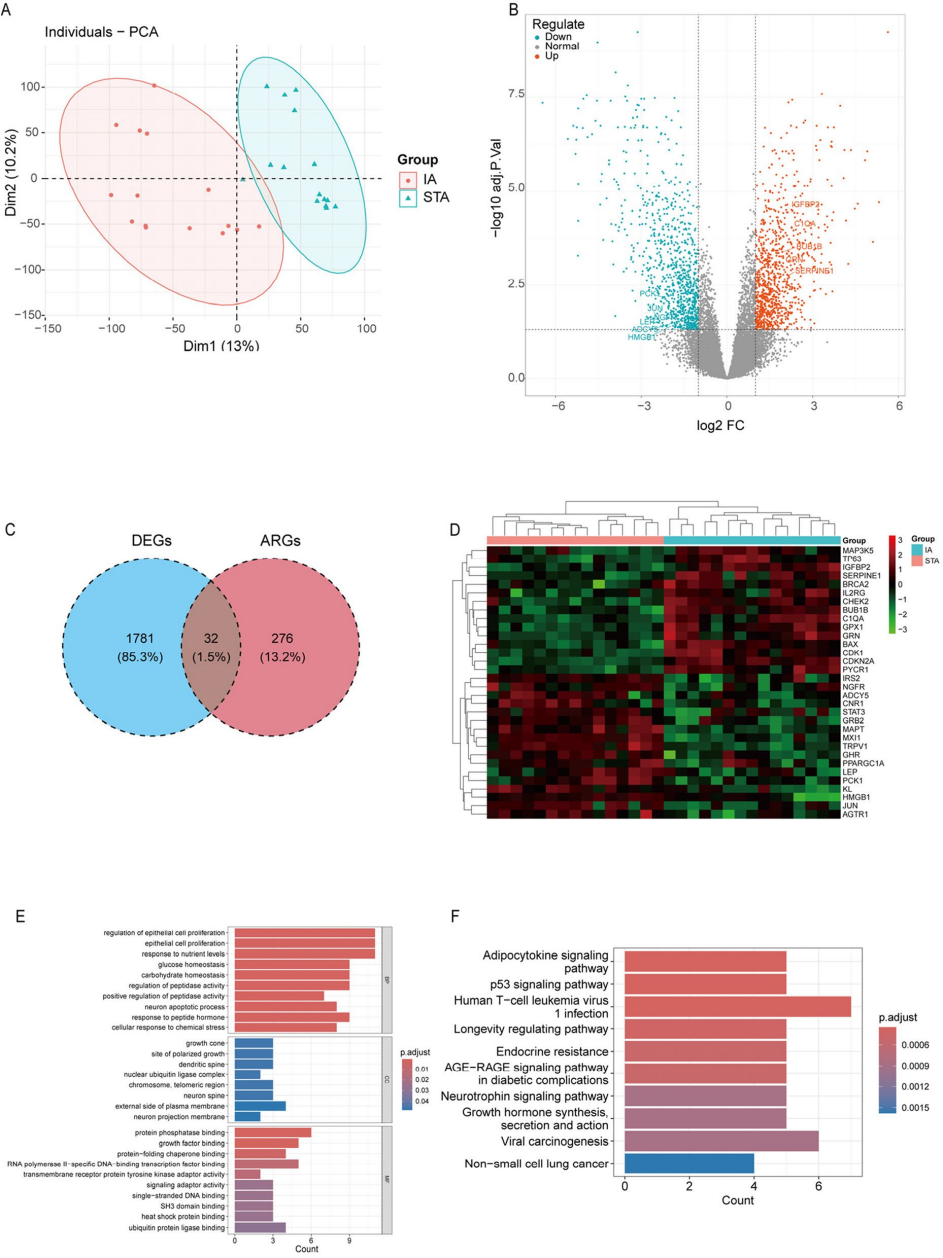
**A** PCA between the IA group and STA group. **B** Volcanic diagram of differentially expressed genes. **C** A Venn diagram showing the intersection of DEGs and ARGs. **D** A heatmap of 32 DEARGs. **E** and **F** GO and KEGG enrichment analysis results of DEARGs

The design of this study is as shown in Fig. 1. Based on the expression profile of GSE75436, principal components analysis (PCA) showed that the IA group and STA group were significantly distinguished at the gene expression level (Fig. 2A). Differential expression analysis showed that there were 1812 DEGs in common between the IA and STA groups, of which 933 upregulated genes and 879 downregulated genes. The distribution of genes was displayed using a volcano plot (Fig. 2B). After taking the intersection of DEGs and 307 Human aging-related genes obtained from the HAGR database, 32 DEARGs were identified (Fig. 2C). A heatmap was generated for DEARGs, including 15 upregulated genes and 17 downregulated genes (Fig. 2D).

### Enrichment analysis

To further explore the potential role of DEARGs in the occurrence of IA. Perform GO and KEGG enrichment analysis on 32 DEARGs to explore their potential biological functions and signaling pathways. The GO analysis results indicate that these genes are mainly enriched in biological processes such as regulation of epithelial cell proliferation, epithelial cell proliferation, carbohydrate homeostasis, regulation of peptidase activity, and positive regulation of peptidase activity. Cellular components include growth cone, nuclear ubiquitin ligase complex, chromosome, telomeric region, neuron spine, and external side of plasma membrane. Molecular functions include protein phosphatase binding, growth factor binding, protein-folding chaperone binding, RNA polymerase II-specific DNA-binding transcription factor binding, transmembrane receptor protein tyrosine kinase adaptor activity, heat shock protein binding, and ubiquitin protein ligase binding. KEGG analysis revealed that these genes are closely related to signaling pathways such as adipocytokine signaling pathway, p53 signaling pathway, longevity regulating pathway, endocrine resistance, AGE − RAGE signaling pathway in diabetic complications, neurotrophin signaling pathway, and growth Hormone synthesis, secretion, and action. We visualized the top 10 GO and KEGG enrichment analysis results in the form of bar charts (Fig. 2E and F). These results provide evidence for the potential role of aging-related genes in the formation of IA and suggest that epithelial cell proliferation and regulation, peptide hormone response, regulation of peptidase activity, and metabolic homeostasis may be related to the occurrence of IA. Previous studies have also indicated that metabolism is related to the degeneration of the aneurysm wall [24].

### WGCNA and the identification of candidate hub genes

**Fig. 3:**
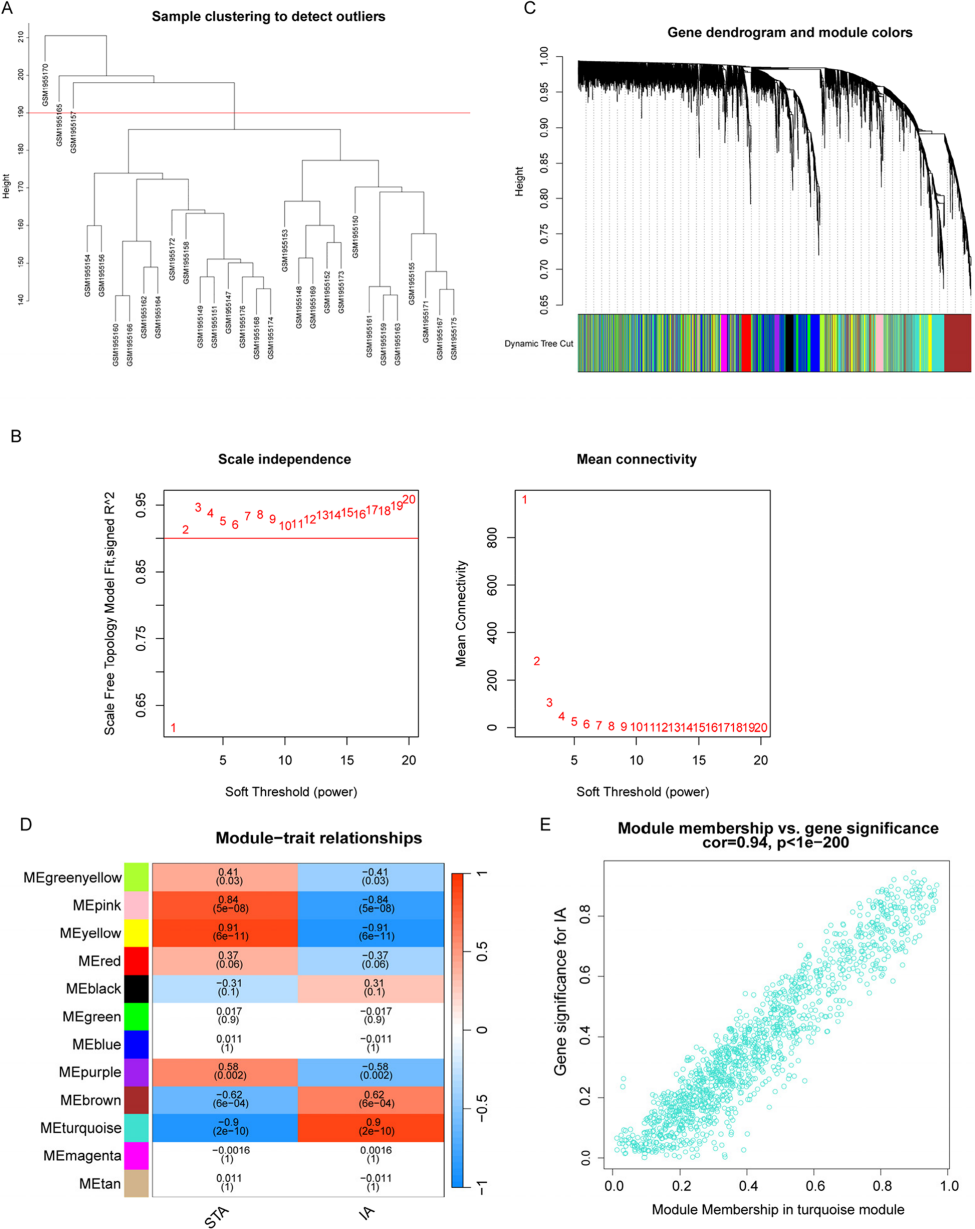
WGCNA analysis. **A** In the sample clustering diagram, remove three outlier samples. **B** Scale-free network construction (power threshold *β* = 4). **C** Generate gene modules from gene tree diagrams. **D** Module and trait correlation heatmap. **E** Scatter plot between the turquoise module and genes related to the module

**Fig. 4:**
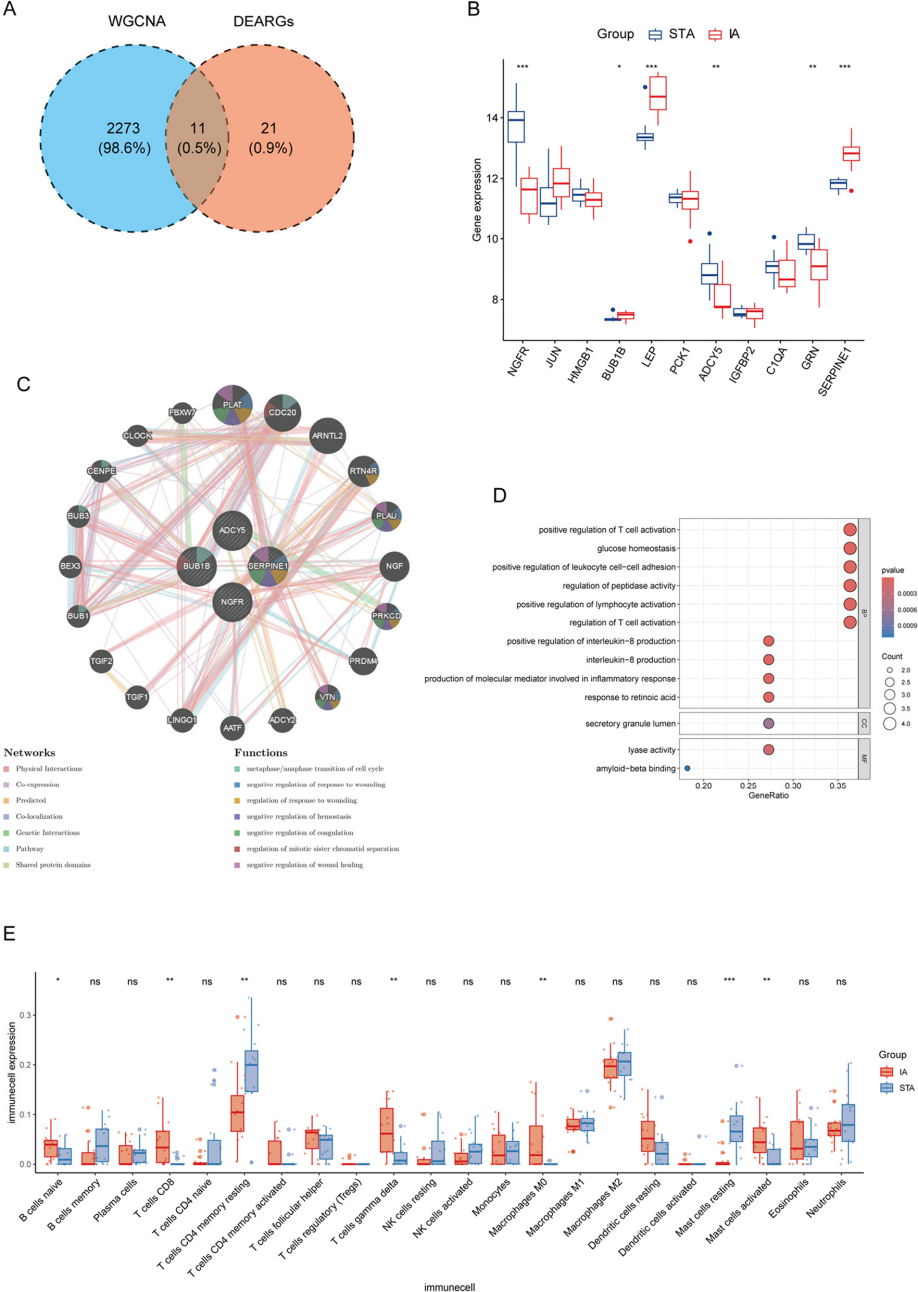
**A** Venn diagram of genes in the turquoise module obtained from WGCNA analysis and DEARGs. **B** Box plots of the 11 candidate hub DEARGs validated in the GSE54083 dataset. **C** The network diagram of co-expressed genes and related functions from GeneMANIA. **D** GO enrichment analysis of co-expressed genes. **E** The CIBERSORT method was used to analyze the differences in immune cell infiltration between IA and STA groups in vascular tissue samples. *p < 0.05, **p < 0.01, ***p < 0.001. ns, *p* > 0.05

We conducted WGCNA analysis based on the gene expression profile of all genes in the GSE75436 dataset. Analyses were performed in accordance with established methodologies from published literature [25]. The sample clustering diagram is shown in Fig. 3A. Choose R^2^ = 0.9 (with a soft threshold of 4) to ensure that the network is scale-free (Fig. 3B). Subsequently, the “cutree Dynamic” function was used to identify co-expressed modules within the network (Fig. 3C); each module contains at least 30 genes, resulting in 12 gene modules (Fig. 3D). Among them, the turquoise module (cor = 0.9, *p* = 2e-10) and the yellow module (cor = − 0.91, *p* = 6e-11) showed the strongest positive and negative correlations with IA, respectively. The turquoise module showed a high positive correlation with the genes related to the module (cor = 0.94, *p* < 1e-200) (Fig. 3E). Analyzing the turquoise module, based on screening criteria (| GS |> 0.20; | MM |> 0.20), we obtained 2284 genes associated with IA phenotype. Finally, these genes were intersected with 32 DEARGs to obtain 11 candidate hub DEARGs (Fig. 4A). Among these candidate hub genes, GRN, BUB1B, IGFBP2, SERPINE1, and C1QA are upregulated, while NGFR, JUN, HMGB1, LEP, PCK1, and ADCY5 are downregulated.

### External verification results

The samples in the validation set GSE54083 dataset were divided into IA and STA groups, and the expression levels of 11 candidate hub DEARGs were extracted. The Wilcoxon test was used to calculate the expression differences between the IA and STA groups. Visualize the results through a box plot (Fig. 4B). The samples in the validation set GSE15629 dataset were divided into IA group and MMA group, and the same method was used for validation. The results indicated significant differences in three genes: NGCR, ADCY5, and SERPINE1. Based on existing research, the four genes that passed validation were identified as the final hub DEARGs, namely NGFR, ADCY5, BUB1B, and SERPINE1. Among them, NGFR and ADCY5 are downregulated, while BUB1B and SERPINE1 are upregulated. Based on GeneCards database (https://www.Genecards.org/), and Table 1 shows their real names and related functions.

**Table 1:** The characteristics and functions of the hub genes

No	Gene symbol	Full name	Function
1	NGFR	Nerve growth factor receptor	Plays an important role in differentiation and survival of specific neuronal populations during development
2	ADCY5	Adenylate cyclase 5	Catalyzes the formation of the signaling molecule cAMP in response to G-protein signaling
3	SERPINE1	Serpin family E member 1	Is a primary inhibitor of tissue-type plasminogen activator (PLAT) and urokinase-type plasminogen activator (PLAU)
4	BUB1B	BUB1 mitotic checkpoint serine/threonine kinase B	Essential component of the mitotic checkpoint

### Co-expression gene network of the hub genes

The co-expression gene network and related functions of the four core genes were analyzed through the GeneMANIA database (Fig. 4C). Subsequently, GO enrichment analysis was performed again on these co-expressed genes (Fig. 4D). The results indicate that the functions of these genes mainly include metaphase/anaphase transition of the cell cycle, negative regulation of response to wounding, regulation of response to wounding, negative regulation of hemostasis, negative regulation of coagulation, regulation of mitotic sister chromatid separation, and negative regulation of wound healing. Co-expressed genes have physical interactions 77.64%, co-expression 8.01%, predicted 5.37%, co-localization 3.63%, genetic interactions 2.87%, pathway 1.88%, and shared protein domains 0.60%.

### Immune cell infiltration

The enrichment analysis results showed that immune-related functions and signaling pathways were activated in the IA group. To further explore the differences in immune cell infiltration in vascular tissue samples between the IA and STA groups, we used the CIBERSORT method to analyze the ratio and expression levels of different immune cells in each sample (Fig. 4E). The results indicated that in aneurysmal tissues, B cells naive, T cells CD8, T cells gamma delta, macrophages M0, and mast cells activated were highly expressed, while T cells CD4 memory resting and mast cells resting were lowly expressed.

### Gene-TF-miRNA network

**Fig. 5:**
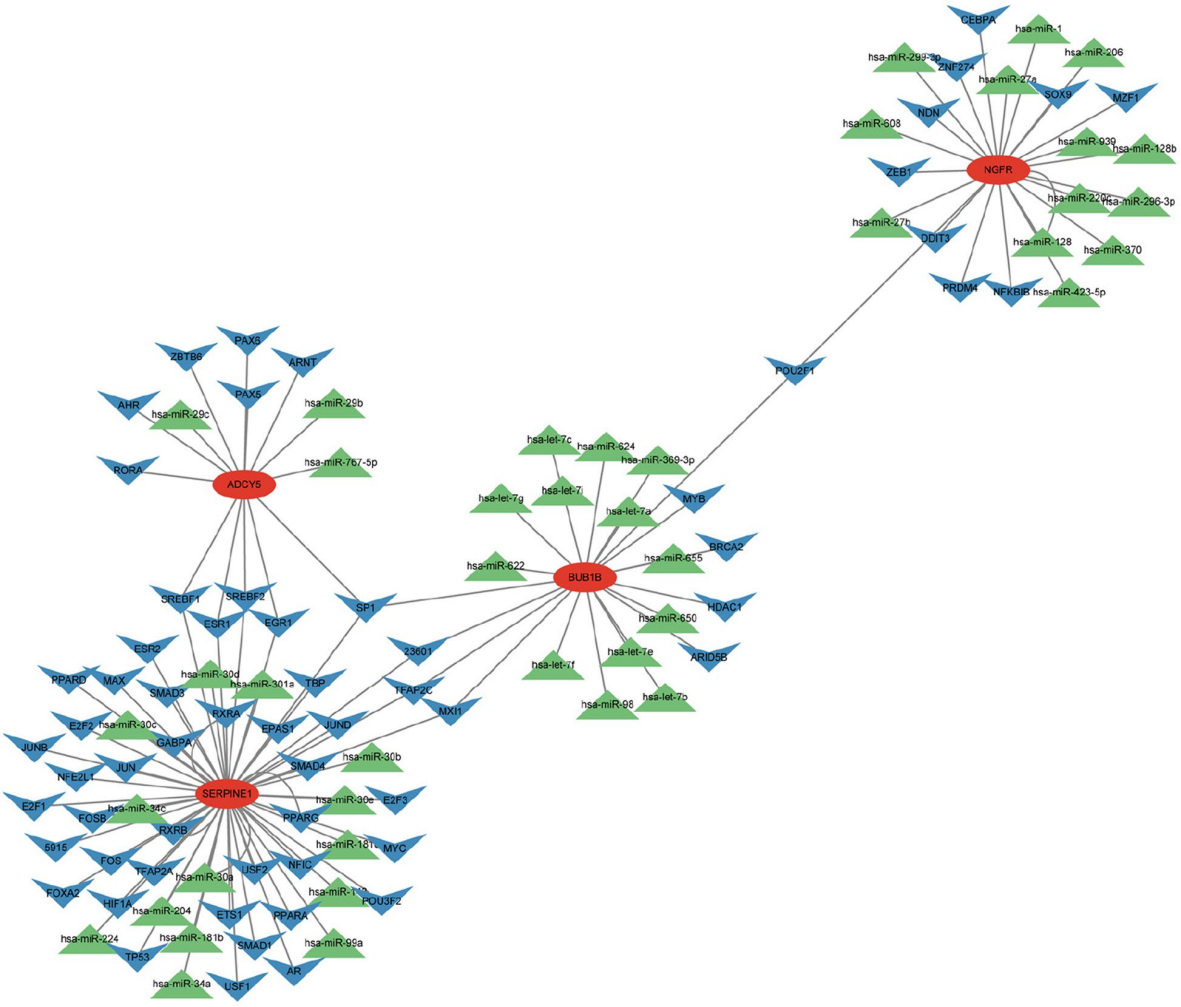
A gene-TF-miRNA regulatory network was constructed. Prediction of upstream TFs and miRNAs for core genes

The NetworkAnalyst online tool was used to predict the upstream transcription factors and miRNAs of the hub genes (Fig. 5). SP1 is a common transcription factor for ADCY5, BUB1B, and SERPINE1, while POU2F1 is shared by NGFR and BUB1B. SREBF1, SREBF2, ESR1, and EGR1 are common transcription factors for ADCY5 and SERPINE1. The SERPINE1 gene has the most predicted miRNAs.

### Drug-gene interactions

**Fig. 6:**
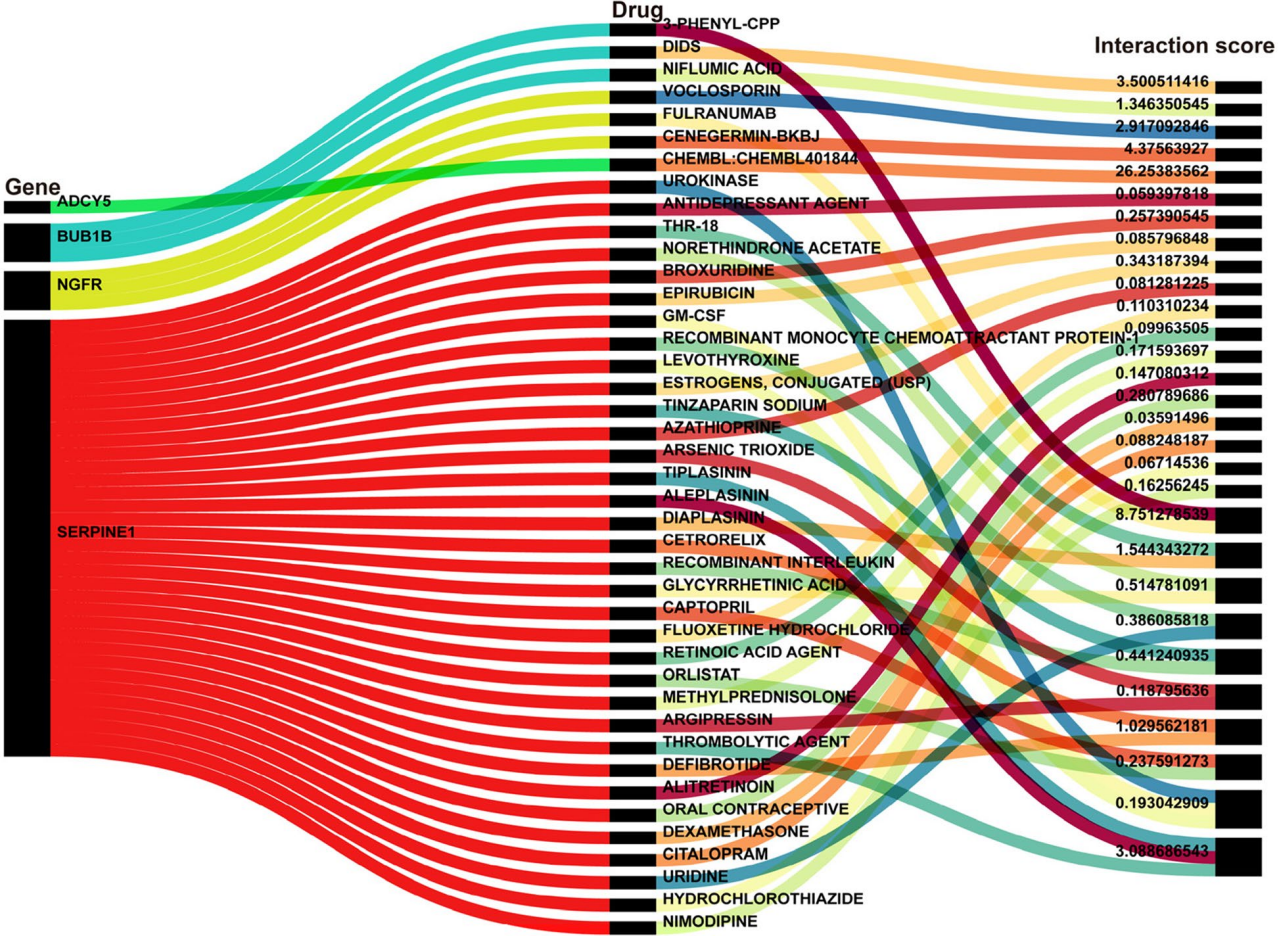
Drug-gene interactions of hub DEARGs

Drug predictions for hub DEARGs were conducted based on the DGIdb database, and the results were visualized (Fig. 6). The results indicate that CHEMBL401844 is a small molecule compound with potential effects on the ADCY5 gene. The NFGR gene has three potential small molecule compounds, among which *fulranumab* is an antibody, *cenegermin-bkbj* is a protein, and *voclosporin* is a small molecule drug with anti-inflammatory and immunosuppressive effects, currently used in the treatment of lupus nephritis. The BUB1B gene has three potential compounds, among which *3-phenyl-CPP* and DIDS are blockers, and *niflumic acid* is a nonsteroidal anti-inflammatory drug. The SERPINE1 gene has 34 potential compounds, among which *tiplasinin*, *aleplasinin*, and *thrombolytic agent* have the highest interaction scores. Based on current basic research and clinical trials, *niflumic acid*, and *voclosporin* show potential in treating vascular inflammation, and further research is warranted.

## Discussion

In recent years, there have been numerous studies on the role of aging-related genes in tumors. However, there are only a few studies on nonneoplastic diseases such as idiopathic pulmonary fibrosis, Alzheimer’s disease, and atherosclerosis [26–28]. Although there is a certain relationship between aging and the occurrence of IA, the specific mechanism of aging-related genes in IA is still unclear.

The aim of this study is to explore the potential role of aging-related genes in the occurrence of intracranial aneurysms. Firstly, we identified 32 differentially expressed aging-related genes by intersecting the differentially expressed genes in intracranial aneurysm tissue samples with Human aging-related genes. Through GO and KEGG enrichment analysis, we understand the biological processes and signaling pathways involved in these genes. Secondly, by intersecting these DEARGs with the co-expressed genes obtained from WGCNA analysis that are highly correlated with IA, we obtained 11 candidate hub DEARGs. These candidate genes are GRN, BUB1B, IGFBP2, SERPINE1, C1QA, NGFR, JUN, HMGB1, LEP, PCK1, and ADCY5. To verify the effectiveness of these genes, the expression levels of these genes were observed in the validation sets GSE54083 and GSE15629, and ultimately four genes were identified as hub genes. These genes include NGFR, ADCY5, BUB1B, and SERPINE1. Among them, NGFR and ADCY5 are downregulated, while BUB1B and SERPINE1 are upregulated.

Nerve growth factor receptor (NGFR), also known as p75NTR, CD271, and TNFRSF16, is a 75-kDa glycoprotein that belongs to the TNF-receptor superfamily [29]. It can bind to NGF or neurotrophic factors with similar affinity to NGF (such as brain-derived nerve factor (BDNF), neurotrophins-3 (NT-3), and neurotrophins 4/5) [30]. Currently, most research on p75NTR mainly focuses on the nervous system and neuronal cells during development or adulthood [31, 32]. p75NTR plays a key role in regulating neuronal growth and cell differentiation [33]. The specific role of NGFR in cell survival or death depends on its ligands and receptors [34]. In mouse experiments, the absence of the p75NTR gene leads to a reduction in dorsal root ganglion neurons, indicating its important role in the development of sensory neurons [35]. NGFR exerts neuroprotective effects by modulating the FGF1/PI3K/AKT signaling pathway [36]. In the animal model of multiple sclerosis (MS), knockout of the p75NTR gene can lead to exacerbated endothelial cell inflammation in the central nervous system and disruption of blood–brain barrier integrity [37]. Bone marrow-specific NGFR knockout exacerbates vascular smooth muscle cell proliferation, promotes neointima formation, and thereby compromises vascular stability [38]. NGFR is functionally linked to neurotrophic factor signaling pathways, and its co-expression network reveals involvement in neurovascular coupling regulation [39]. After spinal cord injury, p75NTR plays a crucial role in neuronal survival and functional recovery [40]. High expression of p75NTR in Schwann cells during peripheral nervous system development and regeneration [41].

Recent studies have shown that p75NTR not only plays a role in neurodegenerative diseases and nervous system injuries but also exhibits different roles in processes such as fibrinolysis, liver repair, and muscle regeneration [42]. After human liver injury, the expression of p75NTR in hepatic stellate cells (HSCs) increases, promoting the differentiation of HSCs into myofibroblasts and thereby regulating hepatocyte proliferation. However, knocking out the p75NTR gene inhibits this process [43]. The expression of NGFR in peripheral blood mononuclear cells (PBMCs) is associated with the progression of vascular remodeling in patients with acute coronary syndrome [44]. Studies have found that NGFR-positive PBMC cells are associated with the severity and poor prognosis of pulmonary arterial hypertension (PAH) [45]. In another study using NGFR gene-deficient mice to construct a hypoxia-induced pulmonary arterial hypertension (PAH) model, it was found that the absence of the NGFR gene can exacerbate hypoxia-induced pulmonary hypertension, causing pulmonary vascular remodeling (characterized by a significant increase in perivascular fibrosis) [46]. The lack of NGFR in lung tissue can increase inflammatory cytokines such as transforming growth factor-beta 1 (TGF-β1), plasminogen activator inhibitor-1 (PAI-1), interleukin-6 (IL-6), and tumor necrosis factor (TNF) [46]. In vascular endothelial cells, NGFR modulates inflammatory responses through NF-κB regulation, while HO-induced inflammatory and metabolic dysfunctions can be ameliorated by suppressing NGFR expression [47]. Some studies have also found that p75NTR is expressed in many immune cells, exists on the surface of immune cells, and can be regulated according to the activation status of cells [48]. However, there is limited research on its specific mechanism and changes in the differentiation and activation process of immune cells [49]. The main histopathological features of intracranial aneurysms include immune inflammatory infiltration, cell death, lipid metabolism, oxidative stress, and protein hydrolysis damage [50]. The infiltration of immune cells is a key factor in the formation and progression of IA [51]. Observation of the tissue remodeling process in animal models of aneurysms [52]. Phenotypic transformation of vascular smooth muscle cells (VSMC) is key to IA vascular wall remodeling [53]. Damage to the arterial wall can induce proliferation of vascular smooth muscle cells and synthesis of new matrix, a process known as intimal hyperplasia [54]. This is also a compensatory response of the body to many degenerative changes during the aging process [55]. The proliferation and matrix synthesis of these vascular wall cells can increase the strength of the aneurysm wall and prevent its rupture. Therefore, based on existing research results, it is speculated that NGFR may play a role in the occurrence of IA by participating in immune cell regulation and vascular wall remodeling.

Adenylate cyclase 5 (ADCY5) is a member of the membrane-bound adenylate cyclase family, which can bind to the adenylate cyclase family and convert adenosine triphosphate into cyclic adenosine monophosphate (cAMP). It synthesizes the metabolic messenger cAMP to mediate G protein-coupled receptor signaling [56]. The ADCY5 gene exhibits pleiotropy and is associated with 2-h glucose challenge, type 2 diabetes, fasting blood sugar, and beta cell insulin secretion [57]. ADCY5 plays a key role in fatty acid oxidation in adipocytes and osteoblasts through cAMP and can regulate the release of free fatty acids into the bloodstream. Its overexpression in adipocytes can promote lipolysis and fatty acid release, while low expression of the ADCY5 gene in osteoblasts can lead to reduced differentiation of osteoblasts [57]. The absence of the ADCY5 gene in mice can reduce overall oxidative stress and extend Lifespan by about 30% [58]; it can also prevent myocardial apoptosis [59], obesity, and insulin resistance [60]. Therefore, it has been proposed to target ADCY5 as a potential therapeutic approach for diabetes, obesity, and cardiovascular diseases [60]. However, some studies have reported that mice with ADCY5 knockout cannot prevent obesity and improve insulin sensitivity [61]. In neurons of ADCY5 gene knockout mice, a decrease in cAMP concentration was observed, leading to increased permeability of the blood–brain barrier [62]. In both the mouse abdominal aortic aneurysm model and human abdominal aortic aneurysm tissue samples, ADCY5 was found to be a differentially expressed gene, and its downregulation is associated with disease progression [63, 64]. The decrease in ADCY5 activity can impair arterial dilation mediated by ADCY5 in diabetic rats [65]. Myocardin (MYOCD) is an important regulator of vascular smooth muscle, and its absence is crucial for IA vascular wall repair and maintenance by regulating VSMC to transition into a proliferative phenotype [66]. The deficiency of ADCY5 can induce smooth muscle cells in the vascular wall tissue of intracranial aneurysms to transform into a proliferative phenotype by downregulating MYOCD, manifested as enhanced cell proliferation, increased extracellular matrix synthesis, and wall attached thrombus formation [67]. Studies have found that the reduced vasorelaxation activity induced by ADCY5 deficiency in IA mice can increase arterial wall tensile strength, leading to an increased survival rate in these mice [67]. The proliferation of vascular smooth muscle cells and endothelial migration are compensatory mechanisms to adapt to increased hemodynamic stress on the arterial wall [68, 69]. These compensatory mechanisms are partially regulated by cytokines released by inflammatory cells infiltrating the vascular wall [70]. The aneurysm wall is also prone to thrombosis formation on the vascular lumen [71]. Myointimal hyperplasia (MH), which refers to the proliferation and migration of vascular smooth muscle cells leading to the formation of a thickened muscular layer on the lumen surface [69]. Vascular wall remodeling is a morphological change caused by myointimal hyperplasia and matrix destruction [68]. The infiltration of T cells and macrophages is related to the rupture of the aneurysm wall, and the infiltration of macrophages is also related to the proliferation of smooth muscle cells in the aneurysm wall [71]. Other studies have found that recurrent sub-adventitial hemorrhage of neovascularization plays a potential role in the pathogenesis of IA, suggesting that IA is also a proliferative disease of the arterial wall [72]. Based on current research, low expression of ADCY5 can promote intimal hyperplasia and matrix synthesis in the vascular wall, which has a certain protective effect on IA. However, further validation through in vivo and in vitro experiments is still needed.

SERPINE1, also known as plasminogen activator inhibitor type 1 (PAI-1), is a serine protease inhibitor. It plays an important role in regulating the plasminogen activation system [73] and can regulate fibrinolysis and promote cellular aging [74]. The SERPINE1 gene encodes plasminogen activator inhibitor type 1 (PAI-1), which in turn antagonizes the activation of plasminogen [75]. It exerts antifibrinolytic effects by combining urokinase-type plasminogen activator and tissue-type plasminogen activator and inactivating them [76]. It is mainly synthesized and secreted by adipocytes, endothelial cells, and platelets [77]. SERPINE1 also participates in biological processes such as protein hydrolysis around cells, tissue remodeling, inflammation, cell migration, and angiogenesis, thus playing a role in various diseases [78]. PAI-1 has a regulatory effect on the contractility of vascular smooth muscle cells. Inhibiting PAI-1 can impair the contractility of VSMCs and significantly alleviate hypertension; thus, SERPINE1 plays a pivotal role in hypertension-related vascular remodeling [79]. High expression of genes related to inflammation, angiogenesis, coagulation, and vascular morphology in human perivascular adipocytes, including SERPINE1 [80]. It has the function of promoting wound repair in human keratinocytes [81], but in tumor cells, it promotes cell proliferation, migration, and metastasis [82]. Urokinase-type plasminogen activator can activate matrix metalloproteinases, leading to the degradation of the extracellular matrix (ECM) and promoting angiogenesis [83]. Reduced extracellular matrix structural proteins were found in the arterial wall of intracranial aneurysm patients [84]. Therefore, genes that maintain the integrity of the extracellular matrix in the arterial wall are associated with the occurrence of intracranial aneurysms [85]. Elevated levels of PAI-1 are associated with coronary artery disease [75] and stroke [86]. Clinical studies on coronary artery disease caused by Kawasaki disease in children have found that patients with coronary artery disease have significantly elevated serum levels of SERPINE1, and the expression of SERPINE1 is significantly upregulated in heart tissue of mouse models and human coronary artery endothelial cells [87]. In cardiovascular disease research, the expression of SERPINE1 in aging fibroblasts significantly increases and inhibits endothelial cell angiogenesis [88]. PAI-1 has a significant inhibitory effect on angiogenesis in myocardial infarction [89]. In patients with cytokine release syndrome (CRS), IL-6 can induce the expression of PAI-1 in vascular endothelial cells, thereby promoting vascular lesions [90]. Another study also suggests that SERPINE1 may be a susceptibility gene for intracranial aneurysms [85]. The polymorphism of the SERPINE1 gene is associated with an increased risk of delayed cerebral ischemia and poor prognosis after aneurysmal subarachnoid hemorrhage [91]. Studies on canine stroke models have found that SERPINE1 is an angiogenesis inhibitor [92]. SERPINE1 expression is upregulated in cerebral endothelial cells during cerebral ischemia–reperfusion injury [93]. Recent studies on ischemic stroke have found that inhibiting the expression of SERPINE1 can promote angiogenesis in brain endothelial cells, thereby increasing cerebral blood flow to the affected area after stroke and preventing brain tissue damage [94]. PAI-1 can directly inhibit endothelial nitric oxide synthase (eNOS) activity and NO production in endothelial cells, leading to endothelial dysfunction and thus inhibiting angiogenesis [95]. Statins can improve the prognosis of ischemic stroke by upregulating eNOS [96] and can also regulate multiple signaling pathways to inhibit PAI-1 production [97]. However, some studies have also shown that SERPINE1 can promote angiogenesis [98]. SERPINE1 expression is elevated in endothelial cells of cerebral arteriovenous malformations, and miR-18a can be used to reduce its level, thereby lowering vascular endothelial growth factor and reducing the formation of abnormal blood vessels [99]. In the study of abdominal aortic aneurysm, it was found that PAI-1 inhibits the activity of matrix metalloproteinases by suppressing the activation of plasminogen [100]. Other studies have also shown that SERPINE1 plays a protective role in the rupture of abdominal aortic aneurysms [101]. The current molecular mechanisms related to SERPINE1 include the following: SERPINE1 can be induced through the TGF-β/Smad signaling pathway [79]. SERPINE1 is a key regulator of ECM remodeling and inflammation [102], capable of inhibiting extracellular matrix proteolysis and cell detachment [103]. SERPINE1 mRNA itself can confer mesenchymal characteristics to cells by sequestering miRNAs, thereby promoting migration and invasion [103]. These studies elucidate the role of SERPINE1 in vascular remodeling, encompassing contractility regulation, ECM remodeling, signal pathway regulation, and other aspects, offering a new perspective for understanding vascular pathophysiology [79]. Therefore, SERPINE1 may influence the formation and rupture of IA by regulating complex mechanisms such as extracellular matrix, tissue remodeling, inflammation, and angiogenesis, which requires further research exploration.

BUB1 mitotic checkpoint serine/threonine kinase B (BUB1B, also known as BUBR1) is a protein encoded by this gene that belongs to the family of spindle assembly checkpoint proteins. It interacts with CDC20 to ensure proper chromosome segregation [104]. It can also inhibit the onset of late stages by suppressing the activation of anaphase-promoting complex or cyclosome (APC/C), ensuring proper chromosome segregation [105]. BUB1B is also involved in DNA repair, neuronal differentiation after mitosis, and ciliary formation [106]. BUB1B plays an important role in SAC signal transduction and stable connection between centromere and spindle microtubules [107]. Therefore, damage to BUB1B and SAC can lead to aneuploidy and chromosomal instability, which may cause cancer progression [108]. Existing research suggests that overexpression of BUB1B is associated with the progression of some cancers [109]. BUB1B is a cancer stem cell-related gene in the study of bladder cancer and lung cancer [110, 111]. However, studies have also shown that high expression of BUB1B can prevent tumor formation [112]. Most studies have shown that high expression of BUB1B is associated with the progression and recurrence of prostate cancer, pancreatic ductal adenocarcinoma, and hepatocellular carcinoma [113–115]. Inhibition of BUB1B kinase activity or reduction of BUB1B levels can lead to chromosome loss and cell apoptosis in human cancer cells [116]. Research on pan cancer has found that changes in the BUB1B gene are closely related to immune cell infiltration. High expression of BUB1B can reduce the infiltration of immune cells and stromal cells and increase the number of tumor cells [117]. However, the specific role and mechanism of BUBR1 in vascular diseases are still unclear. Some studies have found that decreased expression of BUBR1 can affect the vascular system [118, 119]. Mice with low expression of BUBR1 exhibit narrowing of arterial diameter, thinning of arterial wall thickness, reduction of medial VSMCs, arterial wall fibrosis, decreased arterial elasticity, and decreased arterial compliance [118]. Mice with low expression of BUBR1 inhibit endometrial hyperplasia by affecting VSMC proliferation [119]. In limb ischemia experiments, mice with low expression of BUBR1 showed a decrease in vascular endothelial growth factor and impaired recovery of limb blood flow. Therefore, insufficient BUBR1 may impair angiogenesis [120]. Another study found that inhibiting the expression of the BUBR1 gene can alleviate atherosclerosis and macrophage proliferation in hyperlipidemic mice [121]. Therefore, based on existing research results, it is speculated that high expression of BUB1B may increase the stability of the aneurysm wall and prevent its rupture.

In this study, we identified aging genes associated with IA occurrence, providing their potential functions and mechanisms. However, our research also has certain limitations. Firstly, our findings are based on a small public dataset of patients with a relatively small sample size. Although we have validated with external datasets, the pathogenesis associated with these core genes still needs to be validated through more clinical samples and basic experiments. This will become a breakthrough point for our future research.

In summary, we identified the aging-related genes involved in IA through bioinformatics analysis and conducted gene function enrichment analysis. Our research findings suggest that the pathogenic mechanisms of these genes may involve multiple aspects. Especially, NGFR and SERPINE1 may serve as biomarkers or therapeutic targets for IA. This helps us better understand the pathophysiological mechanisms of intracranial aneurysms, discover new drugs or biological therapies, and reduce surgical or invasive treatments.

## Data Availability

No datasets were generated or analysed during the current study.

## Abbreviations

IA: Intracranial aneurysm
VSMCs: Vascular smooth muscle cells
MMP: Matrix metalloproteinase
NOX: NADPH oxidase
GEO: Gene Expression Omnibus
STA: Superficial temporal artery
MMA: Middle meningeal arteries
ARGs: Aging-related genes
KEGG: Kyoto Encyclopedia of Genes and Genomes
WGCNA: Weighted gene co-expression network analysis
GS: Gene significance
MM: Module membership
DEGs: Differentially expressed genes
DEARGs: Differentially expressed age-related genes
TF: Transcription factors
NGFR: Nerve growth factor receptor
BDNF: Brain-derived nerve factor
NT-3: Neurotrophins-3
MS: Multiple sclerosis
HSCs: Hepatic stellate cells
PBMCs: Peripheral blood mononuclear cells
PAH: Pulmonary arterial hypertension
TGF-β1: Transforming growth factor-beta 1
PAI-1: Plasminogen activator inhibitor-1
IL-6: Interleukin-6
TNF: Tumor necrosis factor
VSMC: Vascular smooth muscle cells
ADCY5: Adenylate cyclase 5
cAMP: Cyclic adenosine monophosphate
MYOC: Myocardin
MH: Myointimal hyperplasia
PAI-1: Plasminogen activator inhibitor type 1
CRS: Cytokine release syndrome
eNOS: Endothelial nitric oxide synthase
APC/C: Anaphase-promoting complex or cyclosome
